# Characterizing β-lactam allergy prevalence among patients receiving infectious disease specialty care within a large US healthcare system in Washington

**DOI:** 10.1093/jacamr/dlag033

**Published:** 2026-04-07

**Authors:** Kelly Colas, Jimmy Ma, Kristine F Lan, Anna Berzkalns, Lily Li

**Affiliations:** Division of Allergy and Infectious Diseases, University of Washington, Seattle, WA, USA; Division of Allergy and Infectious Diseases, University of Washington, Seattle, WA, USA; Division of Allergy and Infectious Diseases, University of Washington, Seattle, WA, USA; HIV/STD/HCV Program, Public Health-Seattle & King County, Seattle, WA, USA; Division of Allergy and Infectious Diseases, University of Washington, Seattle, WA, USA

## Abstract

**Background:**

Unverified drug allergies pose a significant barrier to successful antimicrobial stewardship efforts. Most delabelling efforts to date have occurred within an inpatient setting or through outpatient evaluations with an allergy specialist; limited data exist examining β-lactam allergies and delabelling efforts among diverse and underserved populations. Strategies are needed to better identify priority populations for allergy delabelling efforts and the highest-yield opportunities for intervention.

**Objectives:**

We aimed to evaluate the prevalence of penicillin allergy labels (PALs) or cephalosporin allergy labels (CALs) among patients receiving infectious diseases (ID) specialty care across multiple academic and community-based settings and to characterize reported reactions and referral patterns to allergy specialists.

**Methods:**

We conducted a cross-sectional, retrospective cohort study of patients with PAL or CAL receiving care within seven outpatient specialty ID clinics and five inpatient ID consultation services within a large health system between 1 April 2021 and 31 December 2024.

**Patients:**

Inclusion criteria for this study were patients >18 years old with a PAL or CAL listed in the electronic health record who received care at one of the above described clinics during the study time frame.

**Results:**

Among 4951 patients with a reported PAL or CAL, most were male (56%; *n* = 2754), non-Hispanic (86%; *n* = 4242) and white race (53%; *n* = 2623), with a mean age of 48.2 years. PAL and CAL rates were 7.6%–16.7% and 1.1%–5.3%, respectively, in outpatient ID clinics, and 8.8%–15.5% and 3.9%–6.1%, respectively, on inpatient ID consultation services. Of a total of 5965 PAL and 1506 CAL reported entries, most were consistent with hypersensitivity reactions (62.9% and 63.8%, respectively). There were 41.6% of patients with a PAL and 47.7% of patients with a CAL who reported isolated cutaneous symptoms only (flushing, itching, rash or urticaria). Few patients were referred to allergy specialists (7.8%) and ultimately delabelled.

**Conclusions:**

High rates of penicillin and cephalosporin allergy labels, the majority of which reflect low-risk reactions, demonstrate a real ongoing need for innovative strategies to maximize delabelling efforts for patients with unverified drug allergies and infectious care needs.

## Introduction

β-Lactam allergies are commonly reported by up to 10%–20% of the general population.^1,2^ Unverified penicillin allergy labels (PALs) and cephalosporin allergy labels (CALs) are associated with significant clinical and financial consequences.^1,2^ Patients with unverified PALs are more likely to require treatment with second-line antibiotics and experience increased hospital lengths of stay, healthcare costs, drug toxicities and antimicrobial resistance.^1,3^

Evaluation of β-lactam allergy is an important part of antimicrobial stewardship (AMS) programmes and has historically occurred primarily in the hospital setting when there is an imminent need for antibiotics, or through outpatient evaluations with an allergy specialist. Allergy specialty care can be limited in many locations, particularly in rural and underserved communities.^4^ Additionally, the limited data available for demographics and β-lactam allergies suggest that the majority of evaluated individuals are white, well-educated and healthy with few medical comorbidities.^5^ Increasing the availability for evaluation of drug allergy is critical, as over 90% of reported PALs are disproven after additional testing, but only ∼1% of individuals with a PAL currently receive any formal evaluation.^5,6^ Recent data suggest that expanding the scope of drug allergy testing to non-allergists [e.g. internists, paediatricians, clinical pharmacists, infectious diseases (ID) specialists] is a feasible, safe and cost-effective option for β-lactam allergy delabelling.^7,8^ Innovative approaches for evaluating antibiotic allergies across populations are necessary to improve access, health equity, and AMS and other health outcomes.

As the first stage of an effort to expand antimicrobial allergy evaluation and delabelling at the University of Washington (UW), we sought to assess the prevalence of PALs and CALs among patients receiving ID specialty care across multiple academic and community-based settings, and to characterize reported reactions and referral patterns to allergy specialists. In the setting of limited hospital and ambulatory resources, these data can inform potential future opportunities for task-sharing strategies for allergy delabelling and AMS efforts.

## Methods

We conducted a retrospective cohort study of adult patients >18 years old receiving an inpatient ID consultation, care at any outpatient ID or HIV clinic within the UW health system, or seen at the Public Health-Seattle and King County (PHSKC) Sexual Health Clinic between 1 April 2021 and 31 December 2024 (Table S1, available as Supplementary data at *JAC-AMR* Online). We focused on ID-related settings given the frequent complex antimicrobial needs in ID care impacted by β-lactam allergies and the vital part allergy programmes play in AMS efforts. Patient clinical, sociodemographic and allergy data were obtained from the electronic health records (EHRs). This study was approved by the UW Institutional Review Board.

Patients with a PAL or CAL were identified if reported as an active listing in the allergy module of the EHR (Table S2) at the time of the initial clinical encounter. The allergy module of this EHR allows for a brief description of a reaction (selected from a drop-down menu) with an additional ‘comments’ box, in which reaction history can be further described in free text up to 500 characters.

Characteristics of reported PAL and CAL entries and referral patterns to allergists were examined. Descriptive statistics were calculated for PAL and CAL prevalence.

## Results

A total of 4951 patients with a PAL or CAL were identified (Table 1). Overall, most patients with a PAL or CAL reported male gender (56%; *n* = 2754), non-Hispanic ethnicity (86%; *n* = 4242) and white race (53%; *n* = 2623). Mean age at time of evaluation was 48.2 years.

**Table 1.** Patient demographics across outpatient primary and specialty clinics and in-patient infectious disease consult services

Category	All patients evaluated	Patients with allergy	Outpatient clinics								Inpatient services					
			HMC ID Clinic	UW-affiliated Low Barrier Clinics*	Madison Clinic	UWMC-NW ID Clinic	UWMC-ML ID Clinic	Roosevelt Virology Clinic	PHSKC Sexual Health Clinic	All patients evaluated in outpatient ID clinics	HMC General ID Inpatient Service	UWMC General ID Inpatient Service	UWMC NW General ID Inpatient Service	UWMC SOT ID Inpatient Service	UWMC Fred Hutch ID Inpatient Service	All patients evaluated on inpatient services
Unique patients, *n*	40 324	4951	1054	199	572	374	321	61	1507	30 541	730	627	271	279	193	12 054
Age, median [IQR]	40 [30,57]	47 [33,62]	50 [38,60]	44 [37,53]	49 [38,58]	59 [41.25,72]	56 [40,68]	53 [39,62]	32 [27,40]	36 [29,51]	55 [42,67]	58 [41.5,69]	65 [52,76]	57 [46,66]	61 [49,70]	58 [43,68]
Gender, *n* (%)																
Female	12 520 (31)	2140 (43)	396 (38)	115 (58)	126 (22)	225 (60)	209 (65)	18 (30)	463 (31)	8707 (29)	308 (42)	334 (53)	155 (57)	140 (50)	112 (58)	4585 (38)
Male	27 295 (68)	2754 (56)	656 (62)	84 (42)	445 (78)	149 (40)	112 (35)	43 (70)	990 (66)	21 327 (70)	421 (58)	292 (47)	115 (42)	139 (50)	81 (42)	7466 (62)
Non-binary	509 (1)	57 (1)	2 (0)	0 (0))	1 (0)	0 (0))	0 (0))	0 (0))	54 (4)	507 (2)	1 (0)	1 (0)	1 (0)	0 (0))	0 (0))	3 (0)
Ethnicity, *n* (%)																
Hispanic or Latino	5118 (13)	466 (9)	117 (11)	16 (8)	76 (13)	23 (6)	19 (6)	7 (11)	182 (12)	4139 (14)	55 (8)	56 (9)	10 (4)	23 (8)	11 (6)	1279 (11)
Not Hispanic or Latino	32 772 (81)	4242 (86)	894 (85)	162 (81)	469 (82)	325 (87)	264 (82)	49 (80)	1233 (82)	24 190 (79)	665 (91)	560 (89)	259 (96)	251 (90)	181 (94)	10 527 (87)
Not reported	2434 (6)	243 (5)	43 (4)	21 (11)	27 (5)	26 (7)	38 (12)	5 (8)	92 (6)	2212 (7)	10 (1)	11 (2)	2 (1)	5 (2)	1 (1)	248 (2)
Race, *n* (%)																
American Indian or Native Alaskan	632 (2)	92 (2)	23 (2)	5 (3)	15 (3)	1 (0)	6 (2)	1 (2)	18 (1)	393 (1)	19 (3)	17 (3)	2 (1)	9 (3)	2 (1)	304 (3)
Asian	3808 (9)	173 (3)	39 (4)	1 (1)	23 (4)	19 (5)	26 (8)	3 (5)	95 (6)	3033 (10)	25 (3)	40 (6)	8 (3)	17 (6)	17 (9)	918 (8)
Black or African American	5690 (14)	403 (8)	142 (13)	26 (13)	105 (18)	16 (4)	16 (5)	5 (8)	166 (11)	4836 (16)	83 (11)	45 (7)	18 (7)	14 (5)	13 (7)	1160 (10)
Multi-racial	940 (2)	274 (6)	45 (4)	12 (6)	24 (4)	9 (2)	6 (2)	1 (2)	34 (2)	697 (2)	29 (4)	17 (3)	8 (3)	10 (4)	2 (1)	307 (3)
Native Hawaiian or Pacific Islander	844 (2)	64 (1)	10 (1)	4 (2)	4 (1)	1 (0)	4 (1)	1 (2)	29 (2)	692 (2)	5 (1)	5 (1)	1 (0)	5 (2)	4 (2)	183 (2)
White	24 488 (61)	2623 (53)	739 (70)	134 (67)	363 (63)	305 (82)	235 (73)	45 (74)	997 (66)	17 325 (57)	553 (76)	485 (77)	229 (85)	214 (77)	155 (80)	8753 (73)
Another race	394 (1)	1038 (21)	17 (2)	4 (2)	8 (1)	5 (1)	3 (1)	0 (0)	0 (0)	241 (1)	10 (1)	7 (1)	2 (1)	6 (2)	0 (0)	210 (2)
Not reported	3528 (9)	284 (6)	39 (4)	13 (7)	30 (5)	18 (5)	25 (8)	5 (8)	168 (11)	3324 (11)	6 (1)	11 (2)	3 (1)	4 (1)	0 (0)	219 (2)

HMC, Harborview Medical Center; ID, infectious diseases; PHSKC, Public Health-Seattle and King County; SOT, solid organ transplant; UWMC-ML, University of Washington Medical Center-Montlake; UWMC-NW, University of Washington Medical Center-Northwest. *UW- affliated Low Barrier Clinics include outpatient community-health center based walk in clinics (Engage Kent, Engage Federal Way, SHE clinic)

**Figure 1.**
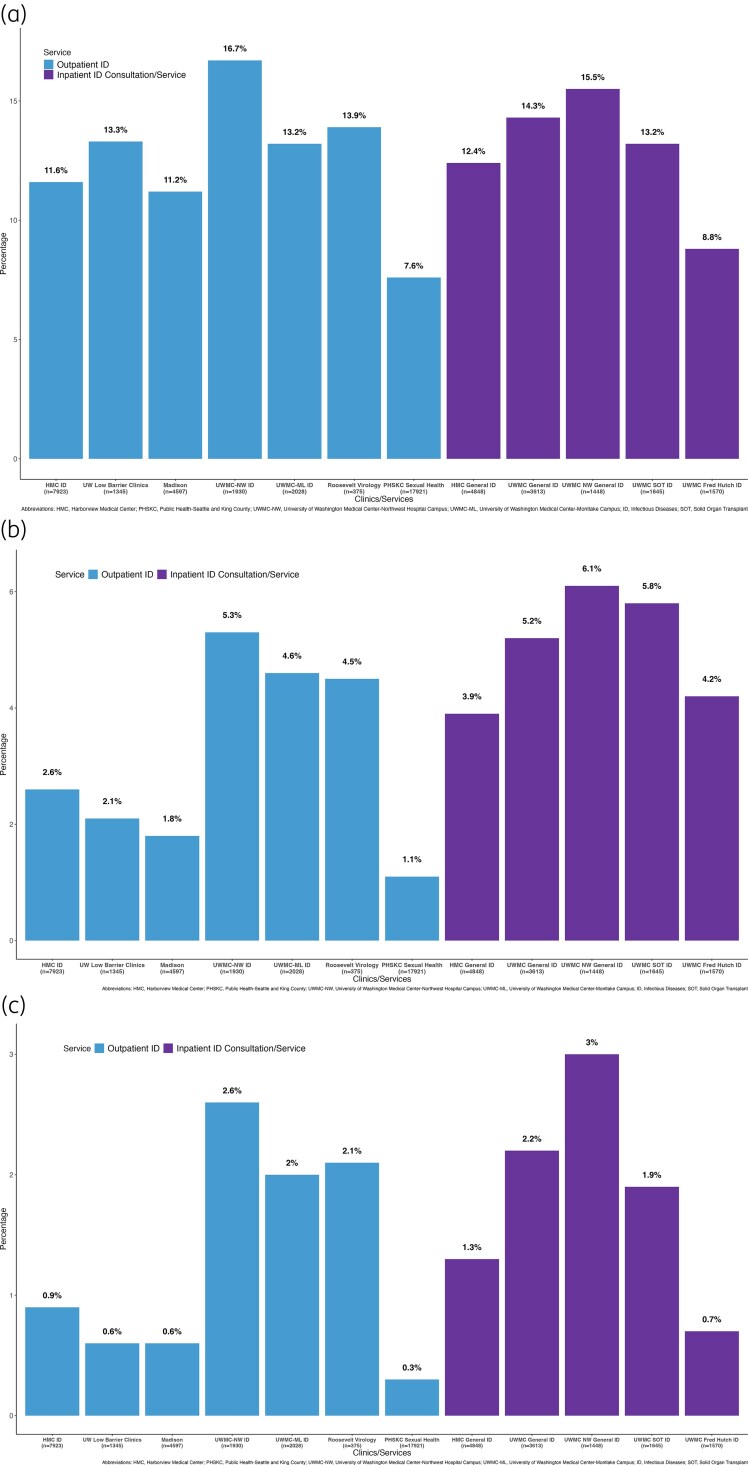
Prevalence of (a) penicillin, (b) cephalosporin, and (c) penicillin and cephalosporin allergy labels among patients receiving care in University of Washington affiliated outpatient infectious disease clinics and inpatient consultation services

Variations in prevalences of PAL and CAL within outpatient and inpatient clinical settings are presented in Figure 1. PAL rates were 7.6%–16.7% in outpatient ID clinics and 8.8%–15.5% on inpatient ID consultation services. CAL prevalence was 1.1%–5.3% in outpatient ID clinics and 3.9%–6.1% of patients evaluated by inpatient ID consultation services. Few patients reported both a PAL and CAL (outpatient ID clinics: 0.3%–2.6%; inpatient ID consultation services: 0.7%–3.0%). In comparison, PAL and CAL prevalences in outpatient non-ID clinics were lower (Figure S1).

A total of 5965 PAL and 1506 CAL allergy reaction entries were reported (Table S3 and S4). Based on reaction descriptions captured in the EHR, 9.2% (*n* = 549) of PAL entries suggested medication side effect or intolerance, and 27.9% (*n* = 1662) were listed as ‘unknown’ or ‘other’ reactions. For cephalosporins, 12.0% (*n* = 181) of reactions were consistent with medication side effects, and 24.2% (*n* = 365) of reactions were reported as ‘unknown’ or ‘other.’ More than half of all reported reactions to penicillins or cephalosporins included symptoms potentially consistent with hypersensitivity (62.9% with *n* = 3754% and 63.7% with *n* = 960, respectively). Notably, 41.6% of all patients with PAL and 47.7% of all patients with CAL reported reactions involving features limited to isolated cutaneous symptoms of flushing, itching, rash or urticaria.

Few patients were referred to an allergist for evaluation of any allergic condition (7.8%), with most patients referred from outpatient clinics. Only 598 patients were delabelled for PAL or CAL during the study time frame, as indicated by a deleted allergy entry.

## Discussion

In this study, we observed a high prevalence of PALs and CALs across multiple ID clinical care settings with diverse populations representing a wide range of clinical and sociodemographic characteristics. The higher prevalence of PALs and CALs in males in this study contrasts with prior studies reporting higher drug allergy prevalence in adult females, likely reflecting the overall distribution of patients evaluated at the included clinics (27 295 males versus 12 520 females),^9^

Estimates of β-lactam allergy prevalence by race are not well established.^5,10^ Most patients with a PAL or CAL reported non-Hispanic ethnicity and white race, consistent with previously published data.^5^ Self-reported black race was the second most common race associated with a PAL or CAL (8%; *n* = 403), with multiracial (6%; *n* = 274) and Asian (3%; *n* = 173) race also represented in our findings. The predominance of male patients with the inclusion of historically underrepresented races in our study population demonstrates a potential unique opportunity for delabelling patients with ongoing antimicrobial needs.

Rates of PAL in outpatient specialty clinics and outpatient ID clinics overall aligned with the national US prevalence of 10%.^1,11^ Notably, the PHSKC Sexual Health Clinic had the lowest reported PAL prevalence (7.6%; *n* = 1507), potentially related to differences in workflow and consistency of allergy label capture during clinical visits. PAL prevalence on inpatient ID services across multiple hospital sites was also overall consistent with the US national average of ∼15%.^6^

Reported CAL prevalence generally aligned with the US average of 1.3%–1.7%, although rates were notably higher in three of the general ID outpatient clinics (4.5%–5.3%; Figure 1b).^2^ This may be related to greater use of cephalosporin antibiotics in the patient populations treated at those clinical sites and variations in clinical practice related to review of drug allergy history at each site. All inpatient ID services demonstrated a higher prevalence of CALs (3.9%–6.1%), which aligns with previous data demonstrating inpatient CAL rates between 4.3% and 6.9%.^12,13^

Approximately 24%–28% of recorded PAL and CAL entries were consistent with medication side effects or intolerances, or listed as ‘unknown/other’. This suggests the potential to delabel around one-fourth of PALs or CALs based on history alone, depending on additional details of reaction history. Notably, over 40% of patients with PAL or CAL allergies had isolated cutaneous symptoms only. Current risk stratification algorithms suggest classification of such patients as low risk and ideal candidates for direct drug challenge and potential delabelling, including by non-allergists,^8,14^

This study illustrates a high prevalence of PAL or CAL across all ID specialty outpatient clinics and inpatient consultation services within one regional academic health system. Although HIV, general ID, transplant and cancer populations have differing infectious risks, resistance patterns and antimicrobial needs, the high prevalence of PALs and CALs suggests similar challenges in removal of unverified antibiotic allergies. Although most PALs are disproven upon further evaluation, barriers such as limited availability of allergists and cost and time constraints for patients may impede completion of formal drug allergy assessments, disproportionately impacting patients already marginalized within the healthcare system.^15^ In the available literature examining outpatient delabelling efforts, studies have demonstrated that a task-sharing strategy of penicillin allergy evaluation with non-allergists is both safe and effective for allergy delabeling,^7,8,16^ Our findings suggest that expanding and tailoring these strategies to specific populations may represent a unique opportunity for patient outreach and targeted delabelling efforts, particularly for those with the highest ID care needs.

Study limitations included the inability to capture allergy or clinical data for patients outside UW/PHSKC and to generalize to other non-ID clinical settings. However, we were able to obtain robust clinical data for a large cohort of patients within diverse ID care settings. Notably, the date of an allergic reaction does not necessarily correlate with the date of allergy entry, indicating that many allergy entries are likely more remote and potentially lower risk for delabelling. Due to the retrospective nature of this study, reaction histories could not be confirmed individually for each patient. There may have been underreporting of drug allergies due to patient recall, manual entry and lack of a standardized, comprehensive review at different clinical sites.

Our study highlights the high prevalence of β-lactam allergies across multiple demographic groups and ID clinic populations, underscoring the clear need to expand creative approaches to assess and evaluate drug allergies tailored for diverse populations with complex antimicrobial needs and often limited access to allergy specialty care. Future research is needed to better understand current barriers and facilitators to targeting the lowest-risk patients for delabelling by non-allergy specialists to more successfully expand outpatient AMS outreach efforts and allow for use of first-line β-lactam antibiotics where indicated.

## Supplementary Material

dlag033_Supplementary_Data

## Supplementary data

Figure S1 and Tables S1–S4 are available as Supplementary data at *JAC-AMR* Online.
