# Supplementary material for: Characterizing β-lactam allergy prevalence among patients receiving infectious disease specialty care within a large US healthcare system in Washington
Source: JAC Antimicrob Resist. 2026 Apr 7;8(2):dlag033. doi: 10.1093/jacamr/dlag033 (PMC13061342; doi:10.1093/jacamr/dlag033)
Supplement: dlag033_Supplementary_Data [file dlag033_supplementary_data.docx]

**Supplemental Material**

**Supplemental Table 1: Clinical outpatient and in-patient sites**

| **Clinical Site** | **Clinic Description** | **Number of patients served during study period** |
| --- | --- | --- |
| **HMC ID Clinic** | **Outpatient general ID clinic** | **7923** |
| **UW-affiliated Low Barrier Clinics** | **Outpatient community-health center based walk-in clinics (Engage Kent, Engage Federal Way, SHE Clinic)** | **1345** |
| **Madison Clinic** | **Outpatient HIV clinic** | **4597** |
| **UWMC-NW ID Clinic** | **Outpatient general ID clinic** | **1930** |
| **UWMC-ML ID Clinic** | **Outpatient general ID clinic** | **2028** |
| **Roosevelt Virology Clinic** | **Outpatient HIV clinic** | **375** |
| **Public Health-Seattle and King Country Sexual Health Clinic** | **Outpatient walk-in sexual health clinic** | **17921** |
| **HMC General ID Inpatient Service** | **Inpatient ID general consult service at Harborview Medical Center** | **4848** |
| **UWMC General ID Inpatient Service** | **Inpatient ID general consult service at UW Montlake** | **3613** |
| **UWMC NW General ID Inpatient Service** | **Inpatient ID general consult service at UW Northwest Hospital** | **1448** |
| **UWMC Solid Organ Transplant ID Inpatient Service** | **Inpatient ID consult service for patients with solid organ transplants at UWMC-ML** | **1645** |
| **UWMC Fred Hutch ID Inpatient Service** | **Inpatient ID consult service for oncology patients at UWMC-ML** | **1570** |
| **Abbreviations: HMC, Harborview Medical Center; ID, infectious diseases; UWMC-NW, University of Washington Medical Center-Northwest; UWMC-ML, University of Washington Medical Center-Montlake; PHSKC, Public Health-Seattle and King County; SOT, Solid Organ Transplant** | | |

**Supplemental Table 2: Penicillin and cephalosporin antibiotic keywords queried within the Allergy module of the electronic health record**

| Antibiotic class | Keywords |
| --- | --- |
| Penicillin | Amoxicillin, Ampicillin, Azocillin, Cloxacillin, Dicloxacillin, Flucloxacillin, Methicillin, Mezlocillin, Nafcillin, Oxacillin, Penicillin, Pipercillin, and Ticarcillin |
| Cephalosporin | Cefaclor, Cefadroxil, Cefalotin, Cefapirin, Cefazolin, Cefdinir, Cefditoren, Cefepime, Cefiderocol, Cefixime, Cefmetazole, Cefoperazone, Cefotaxime, Cefotetan, Cefpodozime, Cefpirome, Cefprozil, Ceftaroline, Ceftazidime, Ceftibuten, Ceftizoxime, Ceftobiprole, Ceftolozane, Ceftriaxone, Cefuroxime, Cephalexin, Cephapirin, Cephradine, and Cefoxitin |

**Supplemental Figure 1.** **Prevalence of A) penicillin, B) cephalosporin, or C) penicillin and cephalosporin allergy labels among patients receiving care in representative University of Washington outpatient general medicine, surgery, or nephrology clinics.**


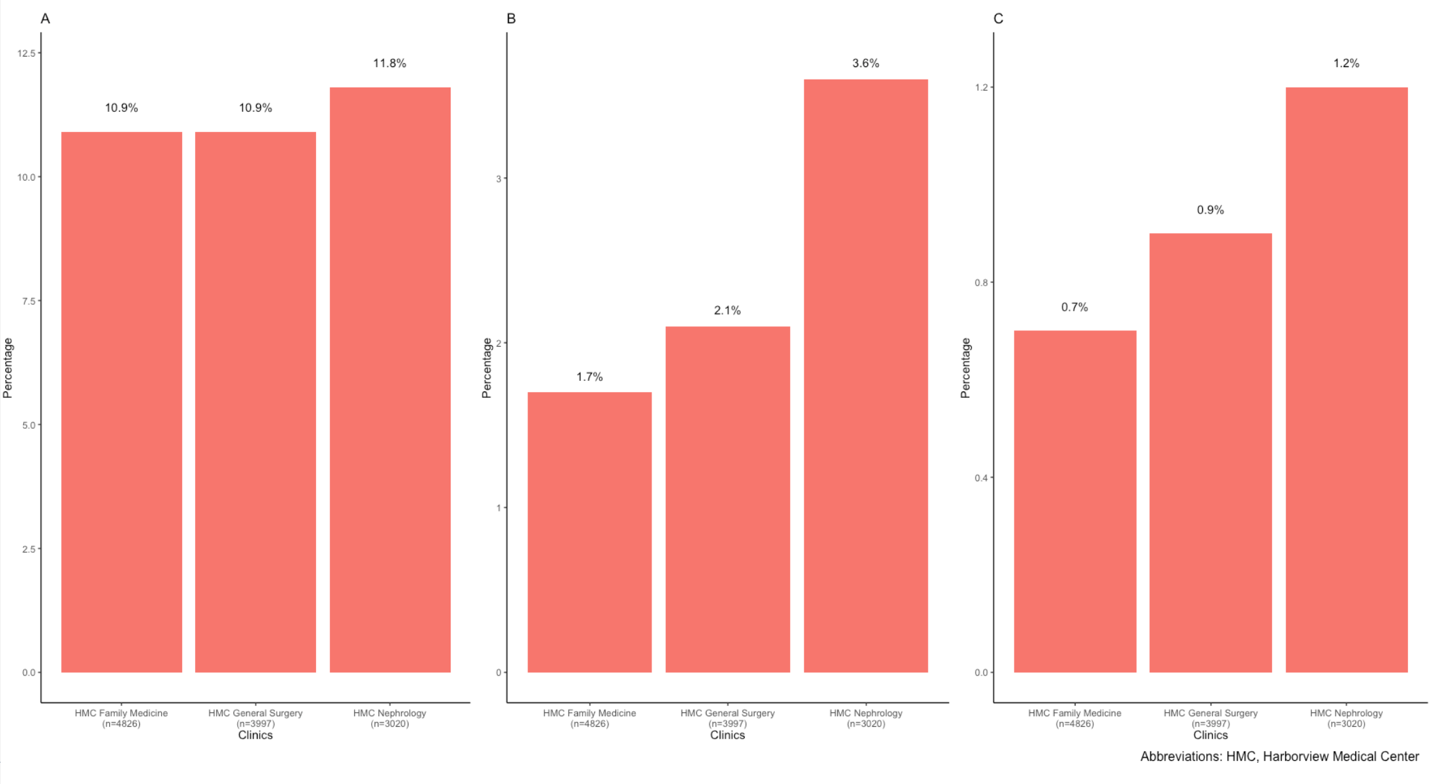


**Supplemental Table 3: Frequency of penicillin reaction types among patients with reported penicillin allergy labels in 5,965 allergy entries* (among 4,273 patients)**

| **Reaction Type** | **No.** | **(%)** |
| --- | --- | --- |
| **Hypersensitivity** | 3754 | 62.9% |
| Anaphylaxis or hypotension | 933 | 15.6% |
| Angioedema or swelling | 370 | 6.2% |
| Bronchospasm, wheeze | 185 | 3.1% |
| Flushing | 59 | 1% |
| Itching/Pruritis | 265 | 4.4% |
| Rash | 1172 | 19.6% |
| Urticaria or hives | 770 | 12.9% |
| **Side effect** | 549 | 9.2% |
| Gastrointestinal upset | 135 | 2.3% |
| Dizziness | 28 | 0.5% |
| Headache | 68 | 1.1% |
| Mental status change | 5 | 0.1% |
| Nausea or vomiting | 303 | 5.1% |
| Loss of Appetite | 10 | 0.2% |
| Other | 1141 | 19.1% |
| Unknown | 521 | 8.7% |
| ***The number of allergy entries exceeds the number of unique patients as each individual may have more than one penicillin antibiotic allergy entry listed in the electronic health record allergy module** | | |

**Supplemental Table 4: Frequency of cephalosporin reaction types among patients with reported cephalosporin allergy labels in 1,506 allergy entries* (among 1,066 patients)**

| **Reaction Type** | **No.** | **(%)** |
| --- | --- | --- |
| **Hypersensitivity** | 960 | 63.7% |
| Anaphylaxis or hypotension | 199 | 13.2% |
| Angioedema or swelling | 68 | 4.5% |
| Bronchospasm, wheeze | 40 | 2.7% |
| Flushing | 16 | 1.1% |
| Itching/Pruritis | 103 | 6.8% |
| Rash | 354 | 23.5% |
| Urticaria or hives | 180 | 12% |
| **Side effect** | 181 | 12% |
| Gastrointestinal upset | 57 | 3.8% |
| Dizziness | 8 | 0.5% |
| Headache | 19 | 1.3% |
| Nausea or vomiting | 96 | 6.4% |
| Loss of Appetite | 1 | 0.1% |
| Other | 277 | 18.4% |
| Unknown | 88 | 5.8% |
| ***The number of allergy entries exceeds the number of unique patients as each individual may have more than one cephalosporin antibiotic allergy entry listed in the electronic health record allergy module** | | |
